# Dies and Taps for Amateurs

**Published:** 1880-01

**Authors:** 


*A Set of Dies and Taps for Amateurs* and others has been sent by the Pratt & Whitney Manufacturing Company of Hartford, Conn., to the writer. For beauty of workmanship and perfection of execution in use, these are superior tools to anything we have seen. Just the thing to enable the amateur to fill in odds and ends of time, if his fondness for machine work is not dormant. Six dies and six taps, and wrench, in a beautiful velvet lined case, ranging from the size of an engine bur shaft to a one-fourth inch bolt. H.

